# Author Correction: Intestinal crypts recover rapidly from focal damage with coordinated motion of stem cells that is impaired by aging

**DOI:** 10.1038/s41598-019-43805-3

**Published:** 2019-09-30

**Authors:** Jiahn Choi, Nikolai Rakhilin, Poornima Gadamsetty, Daniel J. Joe, Tahmineh Tabrizian, Steven M. Lipkin, Derek M. Huffman, Xiling Shen, Nozomi Nishimura

**Affiliations:** 1 Nancy E. and Peter C. Meinig School of Biomedical Engineering, Cornell University, Ithaca, 14853 USA; 2 Department of Biomedical Engineering, Duke University, Durham, North Carolina 27708 USA; 3 Departments of Medicine and Genetic Medicine, Weill Cornell College of Medicine, New York, 10021 USA; 4 Molecular Pharmacology and Medicine, Albert Einstein College of Medicine, Bronx, New York 10461 USA

Correction to: *Scientific Reports* 10.1038/s41598-018-29230-y, published online 20 July 2018

Supplementary material containing SolidWorks files for 3D printing the scaffold was omitted from the original version of this Article. This has been corrected in the HTML version of the Article; the PDF version was correct at time of publication.

