# Supplementary material for: Intestinal crypts recover rapidly from focal damage with coordinated motion of stem cells that is impaired by aging
Source: Sci Rep. 2018 Jul 20;8:10989. doi: 10.1038/s41598-018-29230-y (PMC6054609; doi:10.1038/s41598-018-29230-y)
Supplement: Supplementary file 1 — Supplemental Materials [file 41598_2018_29230_MOESM1_ESM.pdf]

Intestinal crypts recover rapidly from focal damage with coordinated motion of stem cells that is impaired by aging

Jiahn Choi[1], Nikolai Rakhilin[2], Poornima Gadamsetty[1], Daniel J. Joe[1], Tahmineh Tabrizian[4], Steven M. Lipkin[3], Derek M. Huffman[4], Xiling Shen[2], Nozomi Nishimura[1*]

[1]Biomedical Engineering, Cornell University, Ithaca, New York, 14853, USA.
[2]Biomedical Engineering, Duke University, Durham, North Carolina, 27708, USA.
[3]Gastroenterology, Weill Cornell College of Medicine, New York, New York, 10065, USA.
[4]Molecular Pharmacology & Medicine, Albert Einstein College of Medicine, Bronx, New York, 10461, USA.

Correspondence and requests for materials should be addressed to N.N. (email:nn62@cornell.edu)

Supplementary Figure 1

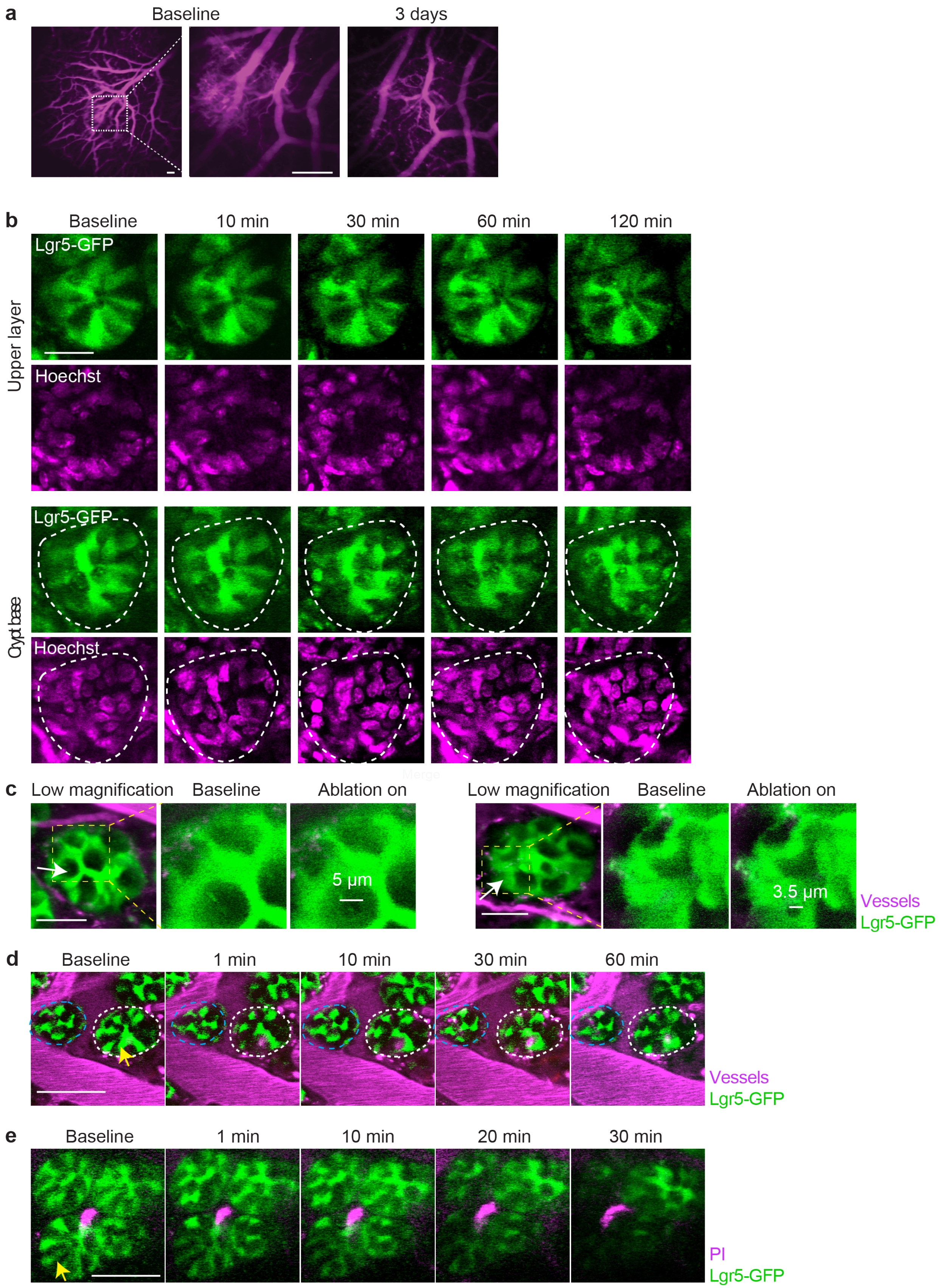

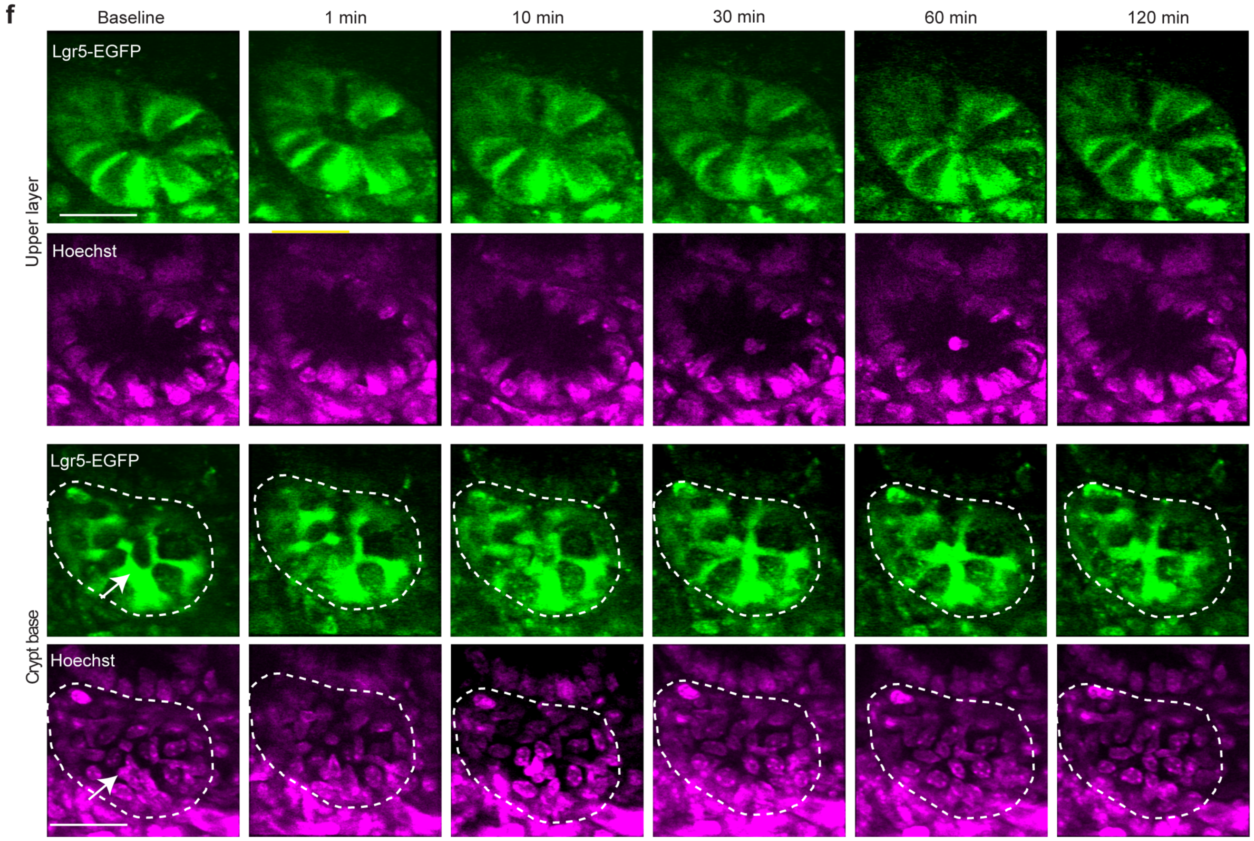

Suppl. Fig. 1. The alternating pattern of the crypt base was rapidly restored after local damage due to femtosecond laser ablation. (**a**) Time-lapse images of small intestinal vasculature at baseline and 3 days showing stability of vasculature over time. To visualize the intestinal vasculature, FITC-dextran (magenta) was injected retro-orbitally. Scale bar: 100 µm. **(b**) Time-lapse images of a crypt in a young mouse labeled with Hoechst with no ablation. This is the same example as Fig. 1e, but the images are separated into two channels to show Lgr5-GFP (green) and Hoechst (magenta). White dash line indicates crypt border. Scale bar: 30 µm. (**c**) Images of crypts during ablation. Yellow dashed line marks the magnified area. When the ablation laser hits, it creates a small hole in the cytoplasm (left: Paneth cell, right: ISC). Green is GFP and magenta shows dye in vasculature. Scale bar: 30 µm. (**d**) Time-lapse images of two adjacent crypts in a Lgr5-GFP mouse. Dashed line indicates each crypt. A yellow arrow indicates focus of laser ablation. Scale bar: 50 µm. (**e**) Attempted ablation with two-photon imaging beam (50s irradiation time, 80-MHz, ~4nJ, 880 nm). Yellow arrow indicates the region scanned by imaging beam at high power. Decline of fluorescent intensity was observed at the targeted crypt and adjacent crypts. This effect is likely both due to the motion of tissue over the long irradiation time as well as the photochemical or thermal nature of the damage. Magenta shows vascular dye. Scale bar: 20 µm. (**f**) Time-lapse images of ablation of one ISC in a Lgr5-GFP mouse displayed with fluorescence channels separated to show Lgr5-GFP (green) and Hoechst (magenta). This is the same example as Fig. 1G and H. White dashed line indicates outline of the crypt. Scale bar: 30 µm.

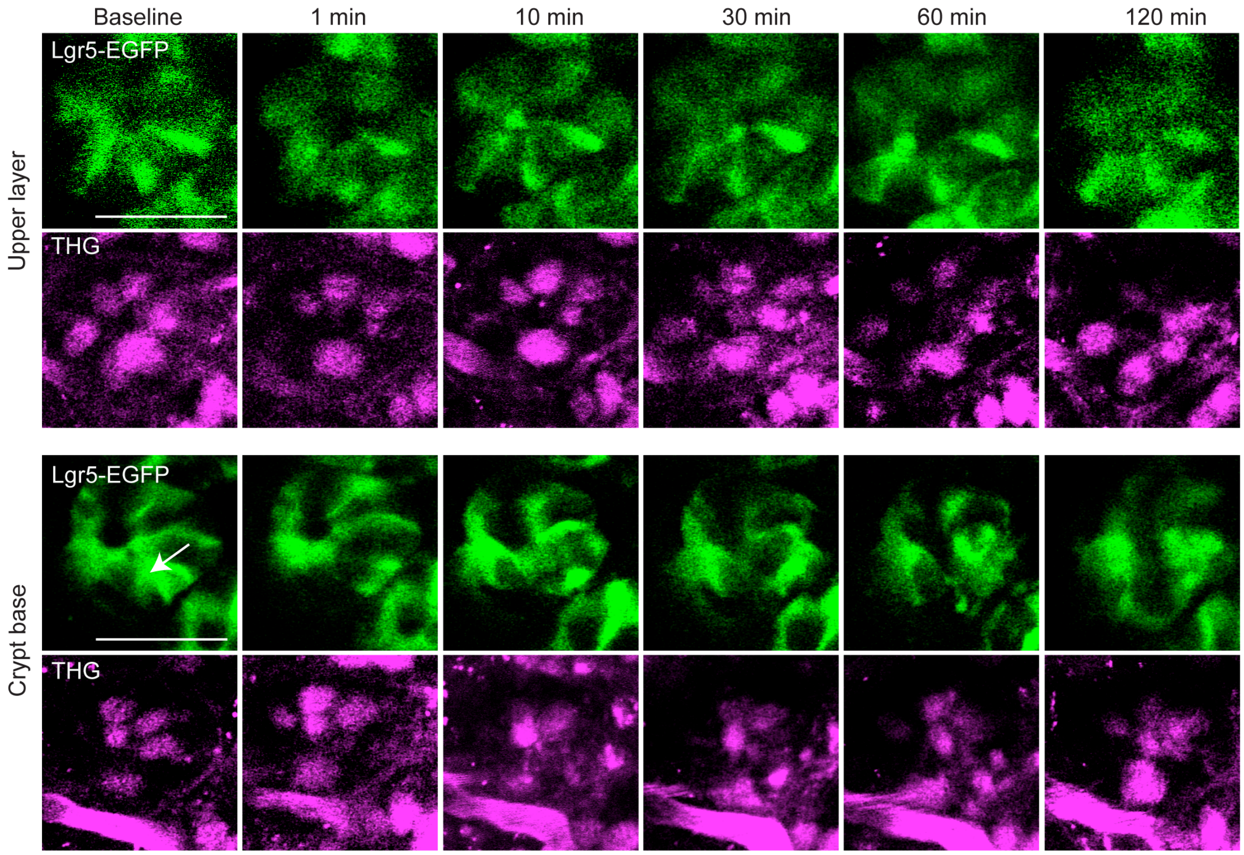

Suppl. Fig. 2. Pattern recovery at the base of the crypt is accomplished by cells residing in the crypt. This is the same example as Fig. 2 shown here with channels separated for clarity. Time-lapse images after ablation of a Lgr5+ ISC in crypt with Lgr5+ ISC (green) and third harmonic generation (THG, magenta). The THG image was taken with 1300 nm wavelength laser excitation and the Lgr5+ GFP image was acquired at 880 nm wavelength. White arrow indicates the area laser focused. Scale bar: 30 µm

Supplementary Figure 3

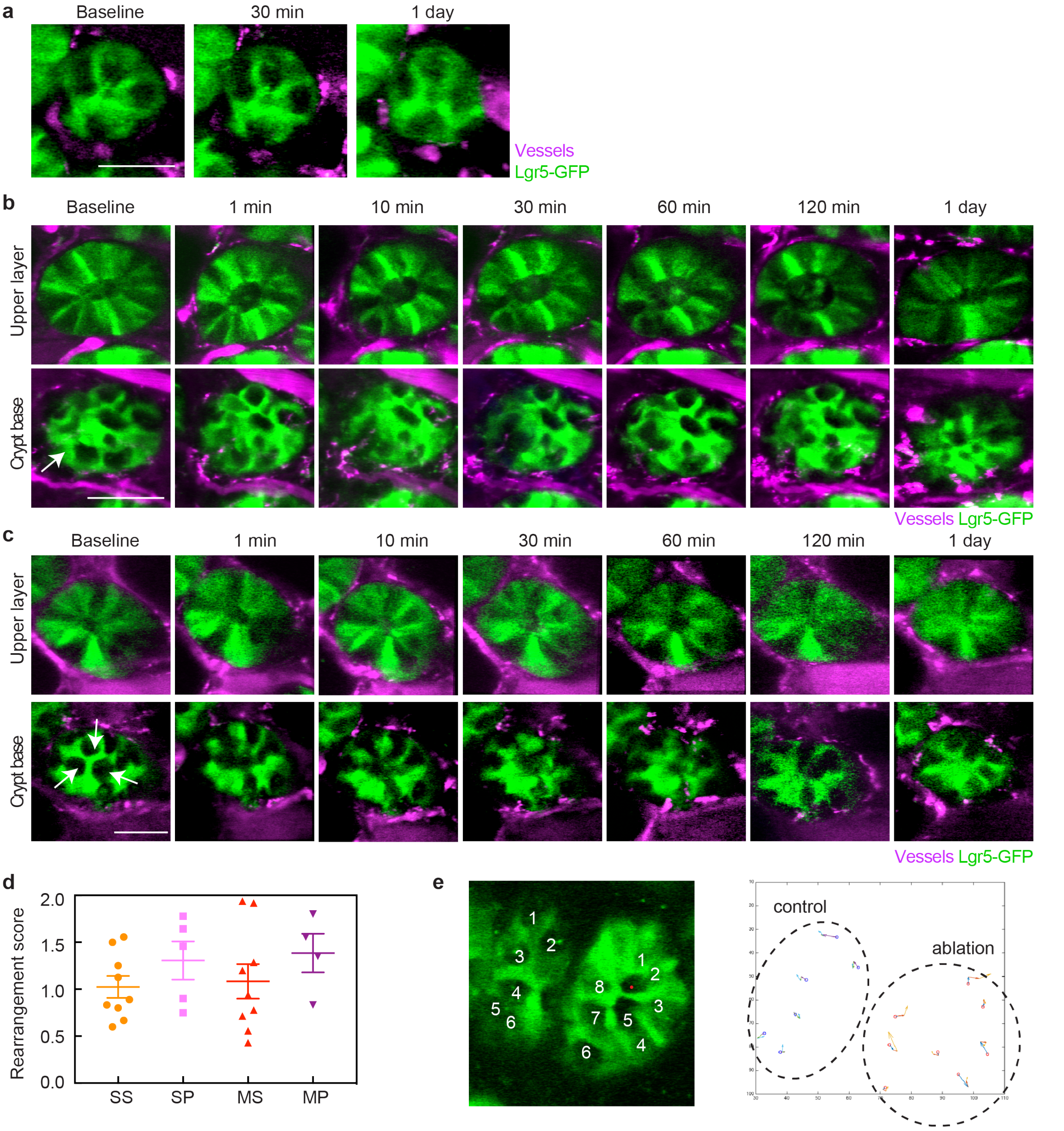

Suppl. Fig. 3. Pattern recovery was accomplished by coordinated motion of crypt cells. (**a**) *In vivo* two-photon microscopy images of a crypt over one day in Lgr5-GFP mouse. Green is GFP. Magenta shows Texas Red in blood vessels and autofluorescence. Scale bar: 20 µm. (**b**) Time-lapse images of crypt with single intestinal stem cell (ISC) ablation. A white arrow indicates ablated Paneth cell. Scale bar: 30 µm. (**c**) Time-lapse images of a crypt with ablation of multiple Paneth cells. White arrows indicate Paneth cells ablated. Scale bar: 30 µm. (**d**) Individual plot of rearrangement score depending on the different types of ablation from Fig. 2d. SS stands for single ISC, SP stands for single Paneth cells, MS stands for multiple

ISCs, and MP stands for multiple Paneth cells. (**e**) Representative example of tracking Paneth cell movement at the crypt base. We numbered each Paneth cell and then got the x-y coordinate from each time point up to 1 hour. The trajectory of Paneth cells in two crypts are shown with open circles representing the starting position at baseline, and vectors representing the new positions at 10, 30 and 60 minutes after ablation.

Supplementary Figure 4

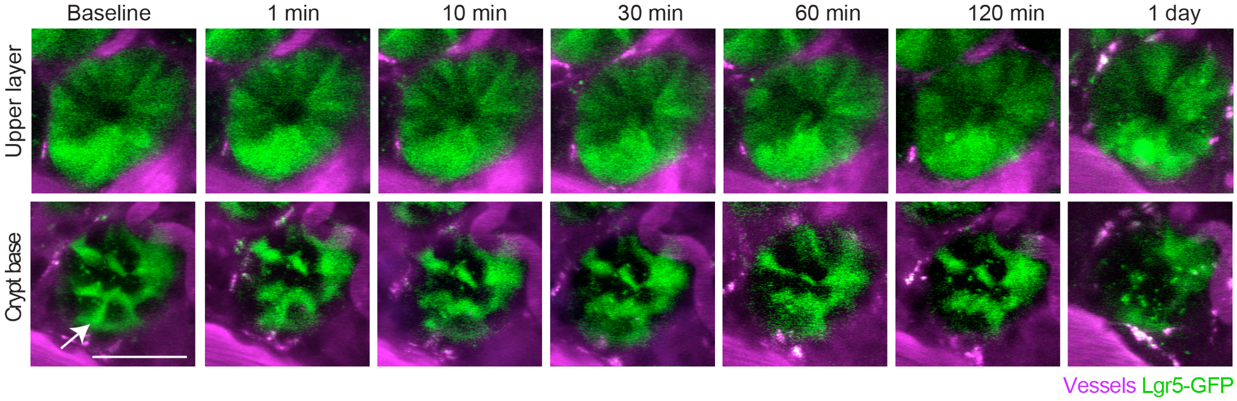

Suppl. Fig. 4. Crypt cell motion is dependent on the ROCK pathway. Time-lapse images of crypt with ablation of single ISC. To inhibit the cellular motility, Y-27632 was topically administered. White arrow indicates a ISC ablated. Green shows GFP and magenta shows dye injected into vasculature as well as some autofluorescence. Scale bar: 30 µm.

***In vivo* imaging**	**Ablation**		**Control**	
Baseline	Unlabeled	THG labeled	Unlabeled	THG labeled
	1.45%	98.55%	1.72%	98.28%
After 2 hours	Unlabeled	THG labeled	Unlabeled	THG labeled
	1.22%	98.78%	0%	100%

***Ex vivo* staining**	Unlabeled	a-Lysozyme labeled	THG labeled	Dual labeled
	0.93%	0.93%	22.22%	75.92%

Suppl. Table 1. Third harmonic generation imaging and immunohistology. THG signal was collected with three-photon microscopy excited wtih 1,300 nm wavelength after ablation of 1 to 3 Lgr5+ ISCs or Paneth cells. 13 crypts in 4 mice were tracked for *in vivo* measurement. For *ex vivo* staining, 16 crypts from 10 different sectioned tissues were examined.

Fig. 1f	Ablation		Control	
crypts (number)	11		5	
mice (number)	4		3	
age	3-10m		3-6m	
sex	F	M	F	M
	3	1	2	1

Fig. 3d, Suppl. Fig. 3d	Ablation		Control	
crypts (number)	28		15	
mice (number)	9		5	
age	2-5m		2-5m	
sex	F	M	F	M
	4	5	2	3

Fig. 3e	Ablation		Fig. 3f	Untreated	
crypts (number)	5		crypts (number)	34	
mice (number)	2		mice (number)	17	
age	3m		age	2-6m	
sex	F	M	sex	F	M
	2	0		10	7

Fig. 5a, b, f	untreated		inhibitor		aged	
crypts (number)	14		24		13	
mice (number)	6		6		5	
age	2-6m		2-6m		17-23m	
sex	F	M	F	M	F	M
	5	1	4	2	0	5

Fig. 5c	untreated		inhibitor		aged	
crypts (number)	22		31		7	
mice (number)	9		9		3	
age	2-6m		2-6m		17-23m	
sex	F	M	F	M	F	M
	9	0	5	4	0	3

Fig. 5d, e	untreated		inhibitor		aged	
crypts (number)	29		28		4	
mice (number)	15		9		3	
age	2-6m		2-6m		17-23m	
sex	F	M	F	M	F	M
	9	6	5	4	0	3

Suppl. Table 2. Age and sex of animals used in statistical analysis.
